# The Silent Morbidity: Impossibilitated Grief and the Erosion of Social Health in Children Exposed to Armed Conflict

**DOI:** 10.7759/cureus.108776

**Published:** 2026-05-13

**Authors:** Luis Mesquita da Fonseca

**Affiliations:** 1 Psychology, Agrupamento de Escolas de Esmoriz - Ovar Norte, Esmoriz, PRT

**Keywords:** armed conflict, child health, impossibilitated grief, neurobiology of stress, social health

## Abstract

In the 2026 landscape of global polycrisis, mass orphanhood in conflict zones has given rise to an underrecognized form of morbidity with profound societal implications. While medical literature has established parameters for prolonged grief disorder, current frameworks often fail to account for contexts in which the structural and relational conditions for mourning are entirely absent. Unlike prolonged grief, which focuses on temporal persistence; absent grief, which implies psychological denial; or disenfranchised grief, which stems from a lack of social validation, impossibilitated grief represents a structural category in which the biological and environmental scaffolds for any mourning process have been decimated. Among children exposed to armed conflict who have lost primary caregivers and lack surviving attachment figures, grief is not merely prolonged but structurally precluded. This phenomenon constitutes a silent morbidity driven by the large-scale interruption of mourning during critical neurodevelopmental periods.

The physiological foundations of this crisis involve sustained neurohumoral stress dysregulation, characterized by persistent activation of the hypothalamic-pituitary-adrenal axis and catecholaminergic surges. In the absence of primary caregivers - who function as external regulators of a child’s stress-response systems - this chronic hypervigilance may erode the biological substrates underlying emotional integration. Furthermore, the destruction of the domestic “holding environment” prevents the development of essential emotion regulation capacities, such as cognitive reappraisal, favoring instead chronic expressive suppression or dissociation.

When experienced at scale, impossibilitated grief contributes to a progressive erosion of social health by undermining the developmental substrates of empathy, trust, and long-term social cooperation. This erosion represents a structural consequence of contemporary conflict, with implications for post-conflict recovery and global stability. Protecting the conditions for mourning must be recognized not only as a humanitarian necessity but also as a critical matter of public health and collective security. The medical community has a responsibility to address the neurobiological futures of children growing up amidst unresolved loss to ensure the long-term stability of future generations.

## Editorial

The contemporary global health landscape is increasingly defined by a state of polycrisis, in which the convergence of protracted armed conflicts, forced displacement, and humanitarian collapse has generated a systemic surge in childhood bereavement [1]. As of early 2026, conservative estimates suggest that more than 1.5 million children living in active conflict zones - including Gaza, Ukraine, and Sudan - have lost one or both primary caregivers [2]. Although established diagnostic criteria for prolonged grief disorder (PGD) exist, such frameworks presuppose the existence of minimally stable relational and environmental conditions that allow mourning to unfold [3]. For many children living in active war zones, these assumptions no longer hold [2,3]. In these contexts, grief is not merely prolonged; it is structurally precluded [4]. This condition must be distinguished from PGD, defined by its duration, or absent and disenfranchised grief, which focuses on internal defenses or external social stigma; impossibilitated grief specifically identifies the total collapse of the relational “holding environment” and safety required for neurobiological integration of loss.

Recent contributions have drawn urgent attention to the destruction of medical communities and healthcare infrastructure under siege [5]. Yet beyond the visible collapse of hospitals lies a secondary failure: the destruction of the domestic and relational “holding environment” required for pediatric survival and emotional development [1,4]. This collapse has contributed to the emergence of the WCNSF designation - a clinical and logistical label for thousands of children who lack surviving relatives able to provide identification, consent, or, critically, biological co-regulation [2]. The epidemiological significance of this population lies not only in their physical vulnerability, but in the systematic interruption of the conditions necessary for mourning itself [2,5].

The physiological foundations of this crisis are profound. Medical literature has documented the neurohumoral features of acute emotional stress, demonstrating how sudden bereavement can precipitate catecholamine-mediated myocardial stunning [1]. This phenomenon offers a mechanistic analogy for understanding the sustained stress physiology experienced by orphaned children in conflict zones [1,2]. If an acute surge of stress hormones can induce transient dysfunction in adults, the prolonged neurohumoral assault faced by children exposed to caregiver loss and ongoing threat may represent a chronic, multisystemic form of morbidity [1]. In the absence of primary caregivers - who function as external regulators of the child’s stress-response systems - the pediatric hypothalamic-pituitary-adrenal axis may remain in a state of persistent hypervigilance [1,4].

From a neurodevelopmental perspective, the interruption of mourning during sensitive periods of brain maturation may profoundly impair the development of emotion regulation capacities [1,3]. In stable environments, mourning rituals facilitate the integration of loss into autobiographical memory [4]. In contexts of persistent threat, however, neural resources are preferentially allocated toward survival-oriented hypervigilance rather than emotional processing [1,2]. This state of suspended grief limits the emergence of regulatory strategies and favors chronic expressive suppression or dissociation. Over time, capacities essential to social health - such as empathy and trust formation - may be attenuated in favor of immediate defensive survival strategies [2,4].

The mass absence of mourning among children in conflict zones is not solely a humanitarian crisis of mental health [2]. When experienced at scale, it represents a structural mechanism contributing to the erosion of social health. Unintegrated developmental trauma becomes embedded within the neurobiological foundations of societies, shaping future patterns of instability [1,4]. Traditional medical diplomacy has rightly focused on the protection of physical infrastructure [5]. However, the restoration of social health requires attention to the psychosocial conditions that allow mourning to occur [3,4]. Protecting the right to mourn is therefore not merely a moral imperative, but a matter of public health and collective security [4]. As the medical community has been called upon to stand by colleagues working under siege [5], it must also consider its responsibility toward the neurobiological futures of children growing up amidst unresolved loss.
